# Seasonal Sleep Variations and Their Association With Meteorological Factors: A Japanese Population Study Using Large-Scale Body Acceleration Data

**DOI:** 10.3389/fdgth.2021.677043

**Published:** 2021-07-02

**Authors:** Li Li, Toru Nakamura, Junichiro Hayano, Yoshiharu Yamamoto

**Affiliations:** ^1^Graduate School of Engineering Science, Osaka University, Toyonaka, Osaka, Japan; ^2^Intasect Communications, Inc., Tokyo, Japan; ^3^Graduate School of Medical Sciences, Nagoya City University, Nagoya, Japan; ^4^Graduate School of Education, The University of Tokyo, Tokyo, Japan

**Keywords:** sleep seasonality, meteorological factors, big data, acceleration data, Japanese

## Abstract

Seasonal changes in meteorological factors [e.g., ambient temperature (*Ta*), humidity, and sunlight] could significantly influence a person's sleep, possibly resulting in the seasonality of sleep properties (timing and quality). However, population-based studies on sleep seasonality or its association with meteorological factors remain limited, especially those using objective sleep data. Japan has clear seasonality with distinctive changes in meteorological variables among seasons, thereby suitable for examining sleep seasonality and the effects of meteorological factors. This study aimed to investigate seasonal variations in sleep properties in a Japanese population (68,604 individuals) and further identify meteorological factors contributing to sleep seasonality. Here we used large-scale objective sleep data estimated from body accelerations by machine learning. Sleep parameters such as total sleep time, sleep latency, sleep efficiency, and wake time after sleep onset demonstrated significant seasonal variations, showing that sleep quality in summer was worse than that in other seasons. While bedtime did not show clear seasonality, get-up time varied seasonally, with a nadir during summer, and positively correlated with the sunrise time. Estimated by the abovementioned sleep parameters, *Ta* had a practically meaningful association with sleep quality, indicating that sleep quality worsened with the increase of *Ta*. This association would partly explain seasonal variations in sleep quality among seasons. In conclusion, *Ta* had a principal role for seasonality in sleep quality, and the sunrise time chiefly determined the get-up time.

## Introduction

Several meteorological factors, such as ambient temperature (*Ta*), humidity, and sunlight, have significant influences on human biological rhythms, including endogenous circadian rhythms (e.g., rectal temperature and melatonin rhythms) and sleep–wake cycles (1–3). Especially, seasonal climatic changes act as rhythmic external cues or perturbations on biological systems that regulate homeostatic and endogenous processes (1, 4, 5). The response of the systems to these seasonal inputs results in seasonal variations of biological variables, such as those of sleep properties.

Seasonal variations in sleep quality or prevalence of insomnia has been well-studied in terms of associations with characteristic seasonal changes in sunlight durations, such as the midnight sun in summer and the dark period in midwinter, especially among Nordic populations. In the epidemiological survey on Norwegian sleep using questionnaires, insomnia was more frequent in winter than in other seasons of the year (6). Other Nordic interview surveys demonstrated that the prevalence of reported insomnia, particularly sleep onset problems, increased from summer to winter in northern Norway but decreased in the southern regions (7). Meanwhile, in a general population in Finland, the prevalence of sleep dissatisfaction increased during summer (8).

Those sleep seasonality are often explained by an entrainment of the circadian time-keeping system to photoperiodic changes (5, 9, 10). However, interestingly, people living in areas with limited daylight variations had significant sleep seasonality (11, 12). For example, in a survey conducted among young Africans living in a dry tropical area, the number of awakenings increased during hot season (11). Furthermore, polysomnography (PSG) revealed that European expatriates living in a similar tropical climate showed seasonal differences in sleep quality and that sleep quality was significantly associated with *Ta* (12). Hence, seasonal sleep variations could not be fully explained by the sole basis of photoperiodic changes among seasons, and sleep seasonality is probably affected by the modulation of thermoregulatory processes passively induced by climatic temperature alterations (5, 11, 12).

Indeed, both laboratory and real-life settings have shown significant *Ta* effects on sleep; a study conducted under a temperature-controlled laboratory reported that *Tas* outside a thermoneutral zone were destructive to sleep (13). Further, subtle manipulations of skin temperature improved sleep latency (SL) in the elderly (14), while sleep depth enhanced in young adults (15), without causing alterations in core body temperatures. Even in a field-based study participated by the elderly with actigraphy, sleep disturbances were significantly related to skin temperature fluctuations (16). Therefore, ambient climate (e.g., bedroom climate), which possibly affects skin temperature, has strong modulating effects on sleep quality (17).

Despite that sleep seasonality and its relationship with meteorological factors have been extensively reported (6–8, 11–13, 17–20), large-scale population studies remain limited. Besides, almost all population studies largely relied on subjective sleep assessments. Though sleep seasonality has been intensively examined in high-latitude countries (e.g., Nordic countries) or low-latitude areas (e.g., tropical areas close to the equator), that in middle-latitude countries (e.g., temperate zone areas) has not been well-elucidated. Furthermore, most studies examined the effects of only a single meteorological factor on sleep, without considering the comprehensive effects of various meteorological factors (e.g., sunrise time, *Ta*, and humidity).

**Figure 1:**
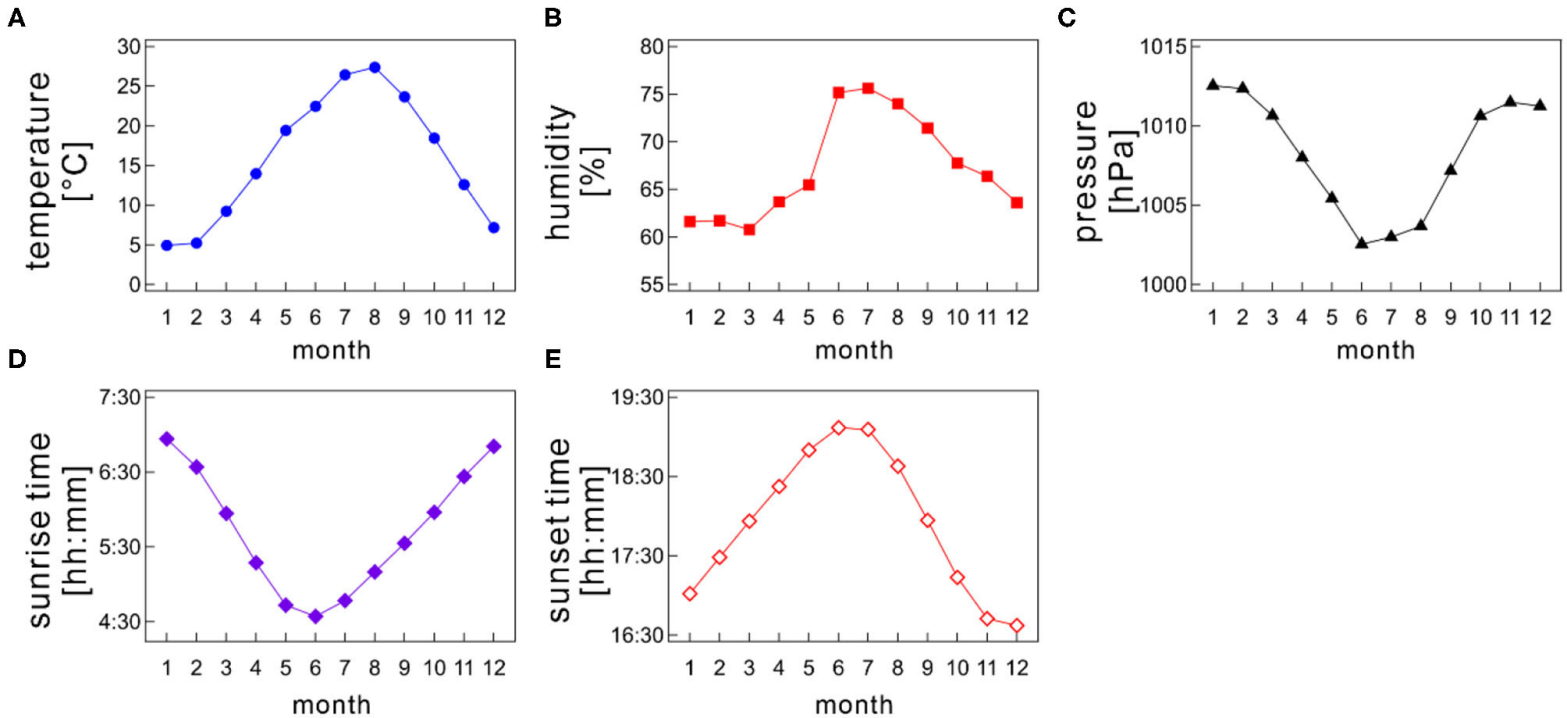
Monthly variations of meteorological variables; **(A)** atmospheric air temperature, **(B)** humidity, **(C)** barometric pressure, **(D)** sunrise time, and **(E)** sunset time as a function of month. The monthly mean values were calculated for each month by averaging the daily meteorological data recorded at a prefectural capital of each medical facility on each Holter recording date.

In examining the seasonal influences of meteorological factors on sleep, Japan is the best location because it is situated in a temperate zone with four distinctive, meteorologically separated seasons (spring, summer, autumn, and winter). Meteorological variables such as *Ta*, humidity, and day length change remarkably among seasons; for instance, in Tokyo, the monthly-based mean atmospheric air temperature varies from a few degrees to roughly 30° throughout a year, and the sunrise time changes from 4:30 AM to 7:00 AM approximately (Figure 1).

Although sleep seasonality is poorly investigated using objective measures in Japanese populations, two distinctive studies have been published (16, 19). An actigraphic study in the elderly reported the decrease of total sleep time (TST) and sleep efficiency (SE) and the increase of SL and wake time after sleep onset (WASO) in summer in comparison with those in winter (16), although the sample size is small. Another sleep study using a contactless biomotion sensor also reported the significant increase of WASO and decrease of SE in summer (19). However, these two previous studies had some inconsistencies in sleep parameter values. For example, the SE in the former study declined ~10% from winter to summer (winter: 91%, summer: 81%), but that in the latter declined slightly (winter: 88%, summer: 86%).

**Figure 2:**
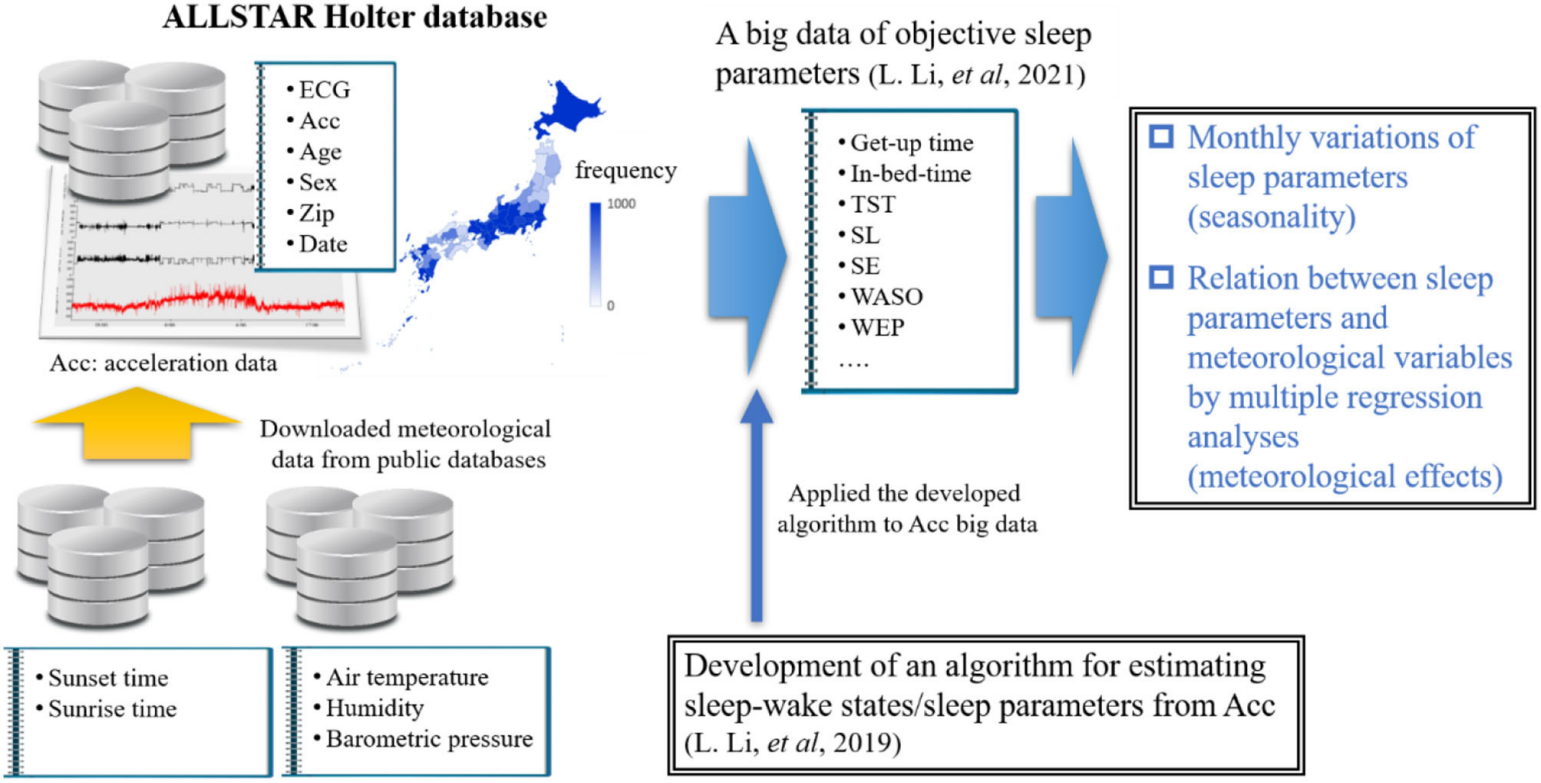
A schematic flow chart of whole research.

Very recently, we examined the effects of age and gender on sleep among Japanese individuals by using a large-scale trunk acceleration data recorded from around 80,000 Japan residents (21, 22) (Figure 2). In that study, we developed an algorithm to determine sleep–wake states from the acceleration data using machine learning approaches and then obtained objective sleep parameters (e.g., sleep duration and SE). The present study aimed to examine the seasonal variations of sleep parameters in a Japanese population by using large-scale objective sleep data and to identity which meteorological factor significantly contributed to seasonal variations in each sleep parameter, if they exist, by multiple regression analysis combined with a bootstrapping method. In other words, this study is a comprehensive sleep research that used objective sleep measures to examine the effects of various ambient meteorological factors on Japanese habitual sleep at the population level.

## Materials and Methods

### Acceleration Database—ALLSTAR Research Project

We used a database constructed by the ALLSTAR research project (23–25). The ALLSTAR database has been thoroughly explained elsewhere (23–25). Briefly, the database stores 24-h electrocardiography (ECG) data and tri-axial acceleration data measured by Holter recorders (Cardy Series; SUZUKEN Co., Ltd.) for clinical purposes by medical facilities all over Japan (47 prefectures in total). Since November 2007, the database has stored more than 300,000 analyzable ECG data (sampling frequency, 250 Hz) and ~80,000 acceleration data simultaneously measured with ECG (sampling frequency, 31.25 Hz), with accompanying information, including the patient's age and gender, the recording date and time, and location (the medical facility's postal code). Considering that Holter monitoring is generally conducted in natural daily circumstances, not in laboratory settings, over 24 h without any restrictions affecting the patient's daily activities, we can access the patient's physiological data (e.g., acceleration data) during habitual sleep.

### Samples

The dataset we used is the same as that reported in our previous study (22). We utilized 68,604 individual acceleration data (30,485 males, 37,951 females, and 168 individuals with unknown gender; age range: 10–89 years old; data length > 20 h) gathered from 2010 to 2016 across Japan. These data were recorded by more than 1,500 medical facilities in 47 prefectures in Japan. Table 1 summarizes age and monthly distributions of the samples. Further, Table 2 shows the mean subjects' age (± standard deviation) stratified by month. The ethics committee of Osaka University approved our study, which conformed to the Declaration of Helsinki.

**Table 1:** Number of samples stratified by month and age group.

**Age group**	**Sample size**	**Month**											
		**1**	**2**	**3**	**4**	**5**	**6**	**7**	**8**	**9**	**10**	**11**	**12**
10s	1,314	70	64	101	103	169	180	160	135	92	84	63	93
20s	1,421	111	109	139	114	133	134	144	95	112	121	89	120
30s	2,816	269	208	234	206	216	245	268	186	204	263	245	272
40s	5,448	507	435	508	411	447	452	454	350	376	478	491	539
50s	7,361	694	674	688	559	596	580	640	458	506	682	668	616
60s	14,727	1,305	1,250	1,457	1,264	1,179	1,219	1,085	858	1,067	1,434	1,363	1,246
70s	21,710	1,856	1,783	2,160	1,824	1,780	1,846	1,698	1,242	1,667	2,172	1,939	1,743
80s	13,806	1,157	1,081	1,283	1,197	1,156	1,227	1,084	856	1,100	1,371	1,233	1,061
Total	68,603	5,969	5,604	6,570	5,678	5,676	5,883	5,533	4,180	5,124	6,605	6,091	5,690

*Note that the recording of month was missed for one subject*.

**Table 2:** Sleep parameter values by month.

**Month**	**Sample number**	**Age**	**TST**	**In-bed time**	**Get-up time**	**SL**	**SE**	**WASO**	**WEP**
		**mean ± SD**	**[min]**	**[hh:mm]**	**[hh:mm]**	**[min]**	**[%]**	**[min]**	**[count]**
1	5,969	66.0 ± 15.7	437.8 ± 1.8	22:19 ± 0:01	6:27 ± 0:01	14.1 ± 0.3	92.4 ± 0.1	36.7 ± 0.6	3.78 ± 0.05
2	5,604	66.3 ± 15.4	430.6 ± 1.8	22:26 ± 0:01	6:27 ± 0:01	13.4 ± 0.3	92.3 ± 0.1	36.7 ± 0.7	3.81 ± 0.05
3	6,570	66.4 ± 15.8	426.2 ± 1.7	22:20 ± 0:01	6:18 ± 0:01	13.8 ± 0.3	92.0 ± 0.1	37.8 ± 0.6	3.94 ± 0.05
4	5,678	66.7 ± 16.1	421.0 ± 1.8	22:17 ± 0:01	5:59 ± 0:01	14.7 ± 0.3	91.7 ± 0.1	39.2 ± 0.7	4.19 ± 0.06
5	5,676	65.6 ± 17.2	409.1 ± 1.8	22:22 ± 0:01	6:08 ± 0:01	15.0 ± 0.3	90.7 ± 0.1	42.5 ± 0.7	4.81 ± 0.06
6	5,883	65.6 ± 17.2	402.6 ± 1.7	22:17 ± 0:01	5:59 ± 0:01	16.0 ± 0.3	90.4 ± 0.1	43.5 ± 0.7	5.24 ± 0.06
7	5,533	64.7 ± 17.5	398.6 ± 1.9	22:23 ± 0:01	6:06 ± 0:01	16.0 ± 0.3	89.3 ± 0.2	48.0 ± 0.7	5.9 ± 0.07
8	4,180	64.9 ± 17.6	404.4 ± 2.2	22:13 ± 0:02	6:02 ± 0:02	15.8 ± 0.3	89.4 ± 0.2	49.0 ± 0.9	6.07 ± 0.08
9	5,124	66.7 ± 16.3	404.7 ± 1.8	22:13 ± 0:01	6:01 ± 0:01	16.4 ± 0.4	89.6 ± 0.2	47.5 ± 0.7	5.67 ± 0.07
10	6,605	66.9 ± 15.6	417.2 ± 1.6	22:15 ± 0:01	6:10 ± 0:01	14.8 ± 0.3	90.7 ± 0.1	43.3 ± 0.7	4.73 ± 0.06
11	6,091	66.7 ± 15.3	425.4 ± 1.7	22:19 ± 0:01	6:17 ± 0:01	14.1 ± 0.3	91.9 ± 0.1	39.0 ± 0.7	4.05 ± 0.05
12	5,690	65.3 ± 16.3	429.7 ± 1.8	22:25 ± 0:01	6:23 ± 0:01	13.4 ± 0.3	92.6 ± 0.1	35.3 ± 0.7	3.69 ± 0.05

*Sleep parameter values are represented as mean ± SEM. SD, standard deviation*.

### Sleep–Wake Inference From the Acceleration Data Using Machine Learning

Sleep and wake states are often inferred according to body movements measured by wearable devices (26–29). Following these approaches, we recently developed algorithms to accurately estimate minute-by-minute sleep–wake states, as well as sleep parameters, from trunk acceleration data measured by the Holter recorder. In this study, we utilized the sleep parameter values calculated by our algorithms in our previous work (21, 22). Our algorithms are summarized below.

Using a support vector machine (SVM), we constructed a sleep–wake classifier (30, 31) that converted tri-axial trunk acceleration data into a sequence of “sleep” and “wake” labels, with 1-min time resolution using the statistical features extracted from the acceleration data. More specifically, we used upper-body tilt angles and local variances of trunk acceleration data as input vectors to the machine. Our method was validated by comparing the outputs of a watch-type sleep monitor (referred to as an actigraph) manufactured by Ambulatory Monitoring Inc. (AMI, Ardsley, NY). An AMI actigraph correctly distinguishes sleep from wakefulness with high accuracy (>90%) (32, 33) and high sensitivity (>95%) (33, 34) compared with PSG, which is the gold standard for sleep assessment. Therefore, the actigraph has been widely used in sleep studies as a PSG substitute (26, 29, 32). Our validation study demonstrated that our SVM-based method was consistent with the AMI actigraph (accuracy = 94.4% ± 3.8%, specificity = 94.2% ± 5.2%, sensitivity = 94.8% ± 3.9%, and F1-score = 92.0 ± 4.5) (21, 22). Note that while we used a classical machine learning approach for the sleep–wake classification, state-of-the-art methods, such as ensemble tree-based algorithms [e.g., extreme gradient boosting (XGBoost) (35), or light gradient boosting machine (LightGBM) (36, 37)], or deep neural networks [e.g., long short-term memory (38–40)], may improve classification performance significantly.

### Sleep Parameters

We examined seasonality of the following seven sleep parameters (22, 28, 29): in-bed time, get-up time, SL, WASO, wake episodes (WEP), TST, and SE. In-bed time is the clock time when a patient gets into bed to sleep and then switches the light off, while get-up time is when a patient finally wakes up in the morning. In-bed time and get-up time are often ascertained by using data from the event marker of an actigraph, sleep diary, or ambient light sensor (29). However, such data were unavailable in the database; hence, we determined those timings from the acceleration data (21, 22). Moreover, SL refers to the time it took a patient to fall asleep; it is the number of minutes between in-bed time and sleep onset, where sleep onset is the time at the start of the first 10 consecutive minutes of sleep after in-bed time. WASO is the sum of the awakening minutes from sleep onset to the get-up time. WEP refers to the number of awakenings between sleep onset and get-up time. TST is the number of minutes asleep between sleep onset and get-up time; it can be calculated by subtracting SL and WASO from time in bed (practically, time in bed was defined by the period between in-bed time and get-up time). Lastly, SE is the ratio of TST to time in bed multiplied by 100. Note that these sleep parameters were strongly related with dynamics in sleep structures commonly assessed by PSG.

### Meteorological Variables

Japan locates in the northern hemisphere, and its climate is separated into four seasons, namely, spring, summer, autumn, and winter. Many meteorological variables, such as *Ta* and photoperiod length, distinctively change among seasons (Figure 1). Each season generally lasts 3 months. Monthly average of atmospheric air temperature is highest during summer (June–August) and lowest during winter (December–February). Meanwhile, spring (March–May) and autumn (September–November) bridge a gap between summer and winter (Figure 1A). Therefore, atmospheric data show an annual sinusoidal pattern. Japan experiences a short rainy season, which generally lasts from the beginning of June to the middle of July, making the area dampish (Figure 1B).

The sunrise time and sunset time also varies between seasons (Figures 1D,E). In summer, the sun rises earlier and sets later, causing a longer daytime; conversely, the sun rises later and sets earlier in winter, resulting in a shorter daytime. Thus, the difference in the daytime length between summer and winter is ~3.5 h. Of note, the daylight-saving time system has not yet been introduced in Japan.

We downloaded daily meteorological data (mean *Ta* [°C], humidity degree (%), and barometric pressure [hPa]) measured in the prefectural capital of each medical facility on each Holter recording date from the open public database of Japan Meteorological Agency (41). Considering that Holter recordings were performed over 2 consecutive days to obtain continuous 24-h data, we used the average *Ta*, humidity, and barometric pressure values over the recording days.

The sunset/sunrise time on the start/end day of the Holter recording was downloaded from the public database of the National Astronomical Observatory of Japan (42).

### Statistics

#### Seasonality of Sleep Parameters

The seasonality (specifically, monthly variations) in each sleep parameter was examined using a generalized linear model (GLM). In fitting a GLM, the month of Holter recording was the categorical variable. Patient's gender and age were also included into the GLM because the gender and age effects were significant in all sleep parameters (21, 22). The age was categorized into eight groups by 10-year intervals (Table 1).

In addition to the main effects of these categorical variables (i.e., age group, gender, and month), the interaction term between age and gender was considered as a possible factor affecting the sleep parameter values. When the interaction term was not significant, a separate GLM without it was created and then fitted to the data again. If the interaction term was significant, we stratified the data by gender or age and then tested simple main effects (i.e., pairwise comparisons) with Bonferroni correction for multiple comparisons. In fitting GLMs, in-bed time and get-up time values were represented as an elapsed time (in minutes) counted from 0:00 on the start day of a Holter recording; hence, the values ranged from 0 to 2,880 (1,440 min × 2 days). Similarly, the sunrise time and sunset time were represented as an elapsed time (in minutes) counted from 0:00 of the start and end day of the recording. The sleep parameter values between July and other months were compared.

All statistical data were analyzed using SAS software version 9.04 (SAS Institute, Cary, NC, USA). In addition, *p*-values were adjusted by Bonferroni adjustment correction for multiple comparisons. To avoid potential inferential biases caused by a large sample size, we considered *p* < 0.01 statistically significant (43, 44). The results were expressed as the mean and the standard error of the mean (SEM) except for the coefficient values in multiple regression analysis explained below.

#### Multiple Linear Regression Analysis

To identify which meteorological variable (i.e., *Ta*, humidity, barometric pressure, sunset time, and sunrise time) contributed to seasonal variations in each sleep parameter, we further evaluated multiple linear regression models in which each sleep parameter was a response variable and meteorological variables were the explanatory variables.

Several meteorological variables highly correlated with each other (e.g., Pearson's correlation was *r* = 0.70 between *Ta* and sunrise time). To avoid the variance inflation caused by high multicollinearity in the regression analysis, we used a shrinkage-based variable selection method, which allowed the exclusion of redundant variables from a regression model. We also combined a model averaging method based on a bootstrapping algorithm [PROC GLMSELECT, ModelAverage (45), in SAS software] to search for a robust and parsimonious model. Each step was explained below in detail.

***Variable selection step*:** Least Absolute Shrinkage and Selection Operator (LASSO) (46), which is a popular method for selecting shrinkage variables, can effectively select important explanatory variables from a set of candidates potentially correlated with a response variable, and then estimate the coefficient values of regressors simultaneously. LASSO belongs to a particular class of penalized least square regression with the sum of absolute values of regression coefficients (or L1 norm), making some coefficients estimated to be zero. In this study, we employed the modified standard LASSO called the adaptive LASSO algorithm (47); in forming the LASSO constraint (i.e., penalized term), weights were applied to each regression coefficient, leading to better performance in identifying a parsimonious model. If all entering explanatory variables were not significant at *p* < 0.01, the selection process was terminated, and from the sequence of models obtained by the selection process, the final model was chosen using the Schwarz Bayesian Criterion (48). Hence, each regression coefficient was ensured to be significant (*p* < 0.01). To adjust both the gender and age effects, we also included the categorical variables of gender and age in the regression models.

***Model averaging step*:** We employed a model averaging method based on a bootstrap method (45, 49) to perform more stable inferences of models. Model selections by the adaptive LASSO regression were repeated on bootstrap samples. The model selected by variable selection possibly varies from sample to sample; therefore, the importance of each explanatory variable was scored by using the number of times it was incorporated in the selected model. Considering that frequently selected explanatory variables were regarded as true underlying regressors, we constructed a final model that used merely the variables above the selection frequency cutoff value. In model averaging, we calculated the ensemble average of each coefficient value estimated by fitting the model to each bootstrap sample. In each bootstrap analysis, 5,000 samples were randomly resampled from the entire dataset. The frequency cutoff value in this study was 70%. Effects of the choice of cutoff values were also examined.

## Results

### Monthly Variations of Sleep Parameters

**Figure 3:**
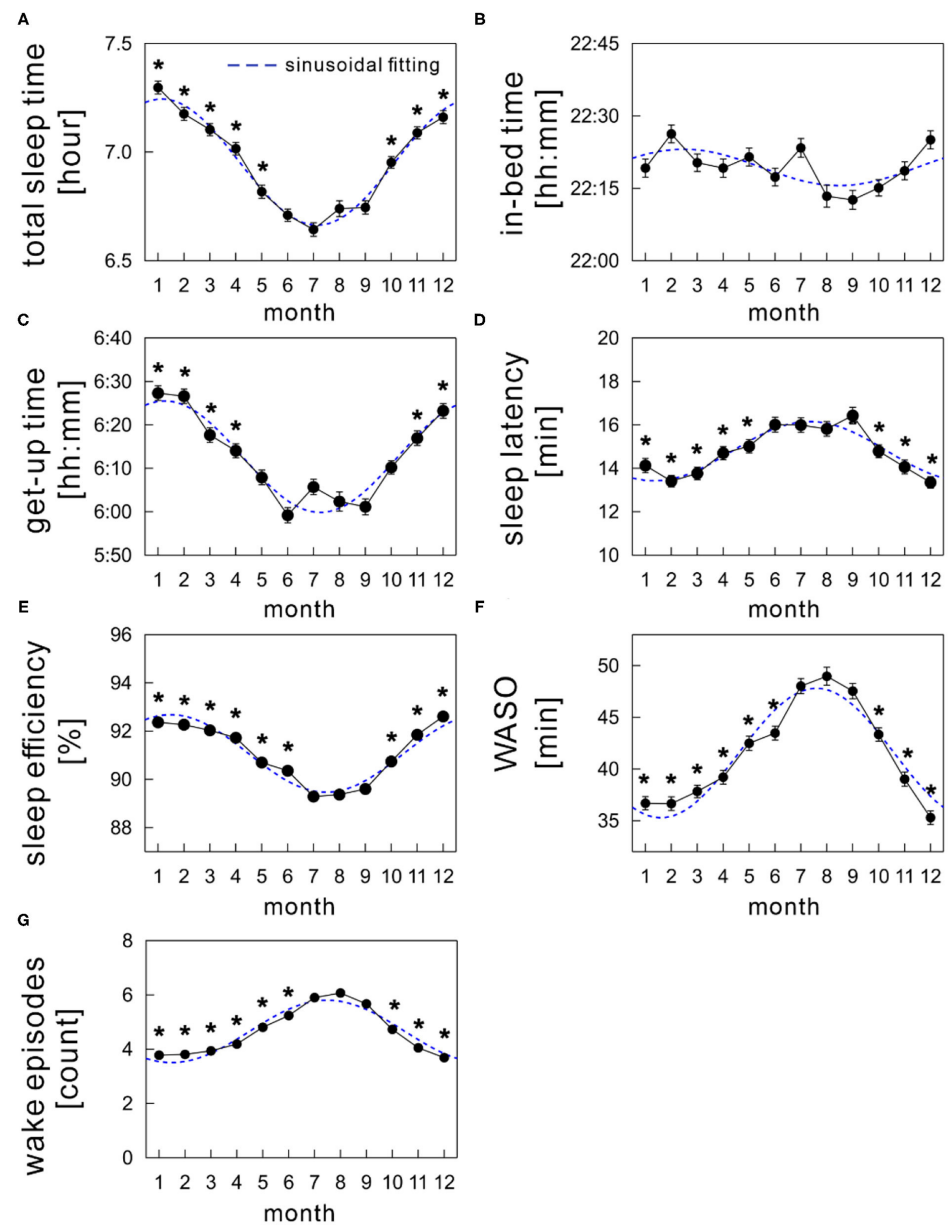
Monthly variations of sleep parameters; **(A)** total sleep time, **(B)** in-bed time, **(C)** get-up time, **(D)** sleep latency, **(E)** sleep efficiency, **(F)** wake time after sleep onset (WASO), and **(G)** wake episodes. The mean values of each sleep parameter are shown as a function of month (solid black circles). The sinusoidal functional curve with 1-year period was fitted to the mean values of each sleep parameter (broken blue curve). The error bars are the standard error of mean. *indicates a significant difference from July (*p* < 0.01).

Figure 3 shows the monthly average values of each sleep parameter (TST, in-bed time, get-up time, SL, SE, WASO, and WEP) as a function of month. The mean TST showed a clear annual cycle with shorter durations during summer and longer durations during winter (Figure 3A). Specifically, it was shortest in July (6.64 ± 0.03 h) and longest in January (7.30 ± 0.03 h), showing a difference of ~40 min monthly.

Seasonal variations were similar in both get-up time (Figure 3C) and SE (Figure 3E), with a nadir in summer. The mean get-up time was earliest in June (5:59 AM) and latest in January (6:27 AM). The mean difference of get-up time between summer and winter was ~24 min (overall mean get-up time: 6:02 AM during summer and 6:26 AM during winter). The mean SE decreased slightly but significantly by ~2.7% in summer compared with that in winter (overall mean SE: 89.7 ± 0.1% during summer and 92.4 ± 0.1% during winter).

The mean in-bed time was almost constant across months; any significant monthly difference was not found between July and other months (Figure 3B). Overall mean in-bed time was 22:19 in our samples.

The monthly average of SL peaked in summer in an annual cycle, although the amplitude of differences among months was subtle (Figure 3D); the mean SL varied between 14.0 and 16.1 min.

Seasonality was noticeable in WASO (Figure 3F). The amount of time of WASO exceedingly increased during summer, with the longest duration of 49.0 ± 0.9 min in August. Conversely, the shortest duration of 35.3 ± 0.7 min was observed in December. Similarly, the number of wake episodes slightly, but significantly, increased during summer compared with the remaining seasons (Figure 3G).

### Meteorological Effects on Sleep Seasonality

**Figure 4:**
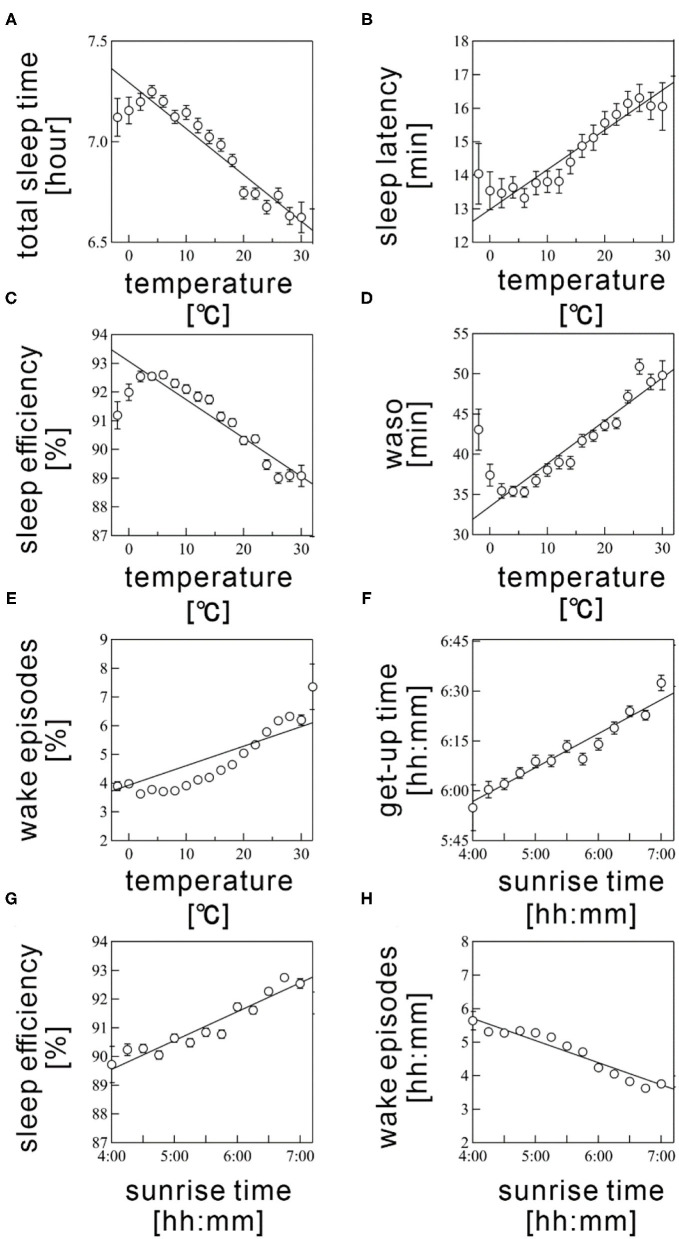
Scatter plots between sleep parameters and meteorological variables. **(A)** total sleep time, **(B)** sleep latency, **(C)** sleep efficiency, **(D)** wake time after sleep onset (WASO), **(E)** wake episodes are shown as a function of *Ta*. **(F)** get-up time, **(G)** sleep efficiency, and **(H)** wake episodes are plotted as a function of sunrise time. Sleep parameter values were averaged every 5°C for *Ta* and 10 min for sunrise time. The error bars are the standard error of mean. The straight line represents a linear regression fit.

According to multiple regression analysis, three meteorological variables, namely, humidity, barometric pressure, and the sunset time, did not significantly contribute to the seasonality of any sleep parameter. Table 3 summarizes the coefficient values of the final averaged model for each sleep parameter. Figure 4 shows the scatter plots between the sleep parameter values and the meteorological variables shown in Table 3. When the frequency cutoff value was changed from 65 to 80%, the final averaged model consistently selected regressors shown in Table 3.

**Table 3:** Coefficient values of the selected significant regressor by multiple regression analysis.

**Sleep parameter**	**Meteorological variable (regressor)**	
	**Ambient temperature [^**°**^C]**	**Sunrise time (elapsed time) [min]**
Get-up time [min]	-	0.18 ± 0.01
TST [min]	−1.58 ± 0.04	-
SL [min]	0.11 ± 0.01	-
SE [%]	−0.111 ± 0.004	0.005 ± 0.001
WASO [min]	0.48 ± 0.01	-
WEP [count]	0.082 ± 0.002	−0.0022 ± 0.0003

*Values are represented as mean ± SD. The humidity, barometric pressure, and sunset time were not selected as significant regressors in any regression model. Therefore, the cells for those regressors are not shown. The bar (-) in a cell indicates that the corresponding regressor was not selected in the final regression model*.

*SE, sleep efficiency; SL, sleep latency; TST, total sleep time; WASO, wake time after sleep onset; WEP, wake episodes*.

The *Ta* was selected as a significant regressor in the final averaged model for all sleep parameters, excepting get-up time. The *Ta* was negatively associated with TST and SE (coefficient value: −1.58 ± 0.04 for TST and −0.111 ± 0.004 for SE; Figures 4A,C) but positively correlated with SL, WASO, and WEP (coefficient value: 0.11 ± 0.01 for SL, 0.48 ± 0.01 for WASO, and 0.082 ± 0.002 for WEP; Figures 4B,D). The linear relations were considerably clear above 5°C (Figures 4A–E). Thus, sleep quality worsened as the *Ta* increased; this result possibly explained the worsening of sleep quality during summer. However, we also found a declining tendency in SE and WASO below 5°C (Figures 4C,D). These suggested a U-shaped correlation of these sleep parameters with *Ta*.

The sunrise time positively associated with get-up time (coefficient value: 0.182 ± 0.006; Figure 4F); this result probably explained the delay of get-up time in winter and the early get-up time in summer. The sunrise time also significantly correlated with SE, though the absolute magnitude of the regression coefficient was practically small (coefficient value: 0.005 ± 0.001); the effect size was below 1% even when the sunrise time changed from 4:30 AM to 7:00 AM. Hence, the sunrise time had practically no influence on SE. As well, the significant, but subtle negative relation was confirmed between sunrise time and WEP. The influence of sunrise time on sleep quality is thought to be limited.

## Discussion

The current study aimed (1) to examine seasonality in various sleep parameters (TST, in-bed time, get-up time, SL, SE, WASO, and WEP) by using a large-scale objective sleep data of a Japanese population and (2) to identify meteorological factors statistically associated with sleep seasonality. This study is the largest population-based research that used objective sleep data in real-life settings to examine sleep seasonality and its association with climatic factors in Japan.

### Seasonality in Sleep Parameters

We found clear seasonal variations with an annual cycle in all sleep parameters, excluding in-bed time. Average monthly values of TST, get-up time, and SE showed a sinusoidal functional form with a nadir in summer, while mean SL, WASO, and WEP peaked during summer. Thus, sleep quality worsened from winter to summer but then improved from summer to winter. These are partly comparable with previous research using objective sleep measures (16, 19), while there are some inconsistencies in sleep parameter values, such as magnitudes of seasonal differences or absolute values of SE. These discrepancies could be influenced by numerous factors, including differences in measurement devices, patients' age and gender distributions, and local climates. Furthermore, the incased frequency in WEP during summer could be related with increased prevalence of self-reported insomnia, especially difficulty in maintaining sleep, in a Japanese population in summer (20).

In our study, seasonal variations were not confirmed in the in-bed time compared with those in the get-up time. Under well-controlled laboratory conditions, both sleep and wake-up times in summer were significantly advanced (5). However, other studies that objectively assessed sleep in real-life settings could not find any seasonal shift in bedtime but wake-up time was significantly advanced during summer (5, 19). Therefore, bedtimes were less influenced by seasonal climate changes in real-life settings. We hypothesized that sociocultural factors (e.g., lifestyle, work, social role, and family) have a large impact on bedtimes in habitual sleep.

### Meteorological Effects on Sleep Seasonality

The most noticeable finding of our study was the identification of meteorological factors contributing to seasonal variations in sleep parameters, using the robust multiple regression analysis. The analysis revealed that *Ta* chiefly determined seasonal variations in sleep quality (TST, SL, SE, WASO, and WEP) in real-life settings in the Japanese population. It would be valuable to address effects of a choice of different classes of sparse regressions. We tested a ridge regression (L2 penalty) (50, 51) and Elastic net (a combination of L1 and L2 penalties) (52). Both methods selected the identical regressors to those of LASSO in the final averaged models at the selection frequencies ranging from 65 to 80%. This indicates the robustness of our results.

The seasonal differences in sleep–wake cycles or sleep quality are commonly interpreted as a consequence of the entrainment of circadian rhythm to photoperiodic changes among seasons (4–7, 53–55). However, interestingly, meaningful contributions of sunlight durations to sleep quality were not detected in our study.

Our results indicated that sleep quality worsened as the *Ta* increases, suggesting the principal role of *Ta* for the seasonality in sleep quality. Further, SE and WASO exhibited a deteriorating trend at colder *Ta* (below 5°C), indicating that sleep quality worsened at colder or hotter *Ta*. These are supported by the results of previous studies based on actigraphy or contactless biomotion sensor (16, 19). Although the functional link between *Ta* and sleep has remained poorly understood, the contribution of a feedback system of skin temperature to sleep-regulating brain areas (preoptic area/anterior hypothalamus) has been suggested as a possible mechanism (56). Indeed, a direct manipulation of skin temperature revealed a notable effect on sleep propensity in the elderly with and without sleep insomnia (14). Without alternating the core temperature, the induction of a small increase (0.4°C) in skin temperature suppressed nocturnal wakefulness and shifted sleep to deeper stages in healthy young and elderly, as well as in patients with insomnia (15). These findings support the interpretation that seasonality in sleep quality was caused by the modulation of skin temperature induced by seasonal changes in *Ta*.

The get-up time did not correlate with *Ta*. This is explained by the difference in the timing of a peak or a nadir in annual cycle of get-up time and *Ta*; the mean get-up time was earliest in June, while the *Ta* was highest in Aug. Meanwhile, the sunrise time had a nadir in June, similar to get-up time. The results of the regression analysis reflect such phase differences between sleep parameters and meteorological variables.

### Limitations

This study has several limitations. The first originates from an ambulatory monitoring in real-life circumstances. Behavioral thermoregulation, such as the use of air conditioning, clothing, and bedspreads, might affect our results because it likely changes both the actual skin and core body temperature. In addition, we did not consider the duration and intensity of light exposure. This limitation could be related to the lack of association between photoperiodic changes and sleep quality. We also did not control the regional differences. Considering that Japan covers several degrees of latitude (from 20 to 46° north), the meteorological variables largely differ between southern and northern areas; for example, the sun rises earlier in northern areas than in southern areas, and monthly *Ta*s are usually lower in northern areas than in southern areas.

Other significant limitations are related to the database. As discussed in our previous study (22), the database probably included selection biases because Holter recordings were usually obtained from patients suspected of having some form of a cardiovascular disease (57). In addition, other clinical conditions (e.g., sleep problems and depression) were not controlled because of the unavailability of such information. The effects of imbalanced age distribution of the samples would be remained. Further population studies controlled those factors might be important. In addition, assessments of sleep structures (e.g., sleep stages) might provide more deeper insights into seasonal influence on nocturnal sleep. Nevertheless, our findings on sleep seasonality derived from the largest Japanese population are scientifically important and informative.

## Data Availability Statement

The data analyzed in this study is subject to the following licenses/restrictions: The database is available for academic research under owners concent. Requests to access these datasets should be directed to http://www.med.nagoya-cu.ac.jp/mededu.dir/allstar/.

## Author Contributions

LL and TN analyzed the data. LL, TN, JH, and YY contributed to manuscript preparation and revision. All authors contributed to data interpretation.

## Conflict of Interest

LL was employed by the company Intersect communications Inc. The remaining authors declare that the research was conducted in the absence of any commercial or financial relationships that could be construed as a potential conflict of interest.
